# Host-cell dependent epigenetic profiles associated with survival outcomes in *T. gondii* infection

**DOI:** 10.1186/s13072-026-00678-x

**Published:** 2026-05-05

**Authors:** Loic Ciampossin, Arzu Ulu, Todd Lenz, Zehao Li, Steven Abel, Sandeep Srivastava, Michael White, Emma Wilson, Karine G. Le Roch

**Affiliations:** 1 Department of Molecular, Cell and Systems Biology, University of California Riverside, 900 University Avenue, Riverside, CA 92521 USA; 2 Division of Biomedical Sciences, University of California Riverside, 900 University Avenue, Riverside, CA 92521 USA

## Abstract

Toxoplasmosis is a widespread parasitic disease affecting roughly one-third of the global population. In immunocompromised individuals or during pregnancy, infection can result in severe complications. Following primary infection, *Toxoplasma gondii* forms dormant bradyzoite cysts in tissues such as the brain and eyes. These cysts can rupture, particularly in immunocompromised hosts, releasing active parasites and triggering recrudescence. Efforts to experimentally induce and study bradyzoite cyst recrudescence have been hindered by the limited capacity of cell culture adapted strains to form tissue cysts in vivo. *T. gondii* employs diverse strategies to persist within host cells, including manipulation of host metabolism and immune responses, and these strategies may vary by host cell type. Here, we profiled epigenomic and transcriptomic features associated with differential parasite survival in distinct cell types. Using an ex vivo model of the Type II ME49 strain unadapted to fibroblast culture, we compared parasite survival and epigenetic profiles in neonatal mouse astrocytes (AST) and human foreskin fibroblasts (HFF). Comparative analyses revealed marked divergence in parasite population dynamics, accompanied by reduced H3K4me3 enrichment at promoter regions in parasites grown in HFF. This epigenetic shift correlated with transcriptomic changes in genes linked to cell cycle progression, growth, and development, including a subset of AP2 transcription factors, underscoring the influence of host cell type on parasite biology.

**Supplementary Information:**

The online version contains supplementary material available at 10.1186/s13072-026-00678-x.

## Introduction

*Toxoplasma gondii* is a highly prevalent apicomplexan parasite that infects roughly one-third of the human population [1]. While infection is often asymptomatic in immunocompetent individuals, toxoplasmosis can cause severe disease in immunocompromised patients and during pregnancy [2, 3]. The parasite alternates between a rapidly replicating tachyzoite stage and a latent bradyzoite stage enclosed in tissue cysts. Chronic infection, maintained by long-lived bradyzoites, is central to pathogenesis and has been linked to neurological, ocular, and inflammatory complications [4, 5]. Despite its clinical relevance, the molecular mechanisms that sustain chronic infection and drive its associated disease outcomes remain poorly understood [6].

In vitro studies of *T. gondii* have relied heavily on human foreskin fibroblasts (HFFs) as a host cell model [7]. While convenient for parasite propagation, HFF cultures provide a limited view of the parasite’s developmental capacity, as long-term passage can drive adaptation that alters gene expression and stage conversion potential [8]. Although cyst formation can occur in fibroblast cultures, it is generally inefficient under standard conditions, and bradyzoite differentiation typically requires artificial induction methods that may not fully replicate the complex cues driving cyst development in vivo. Additionally, prolonged adaptation to fibroblast culture may alter parasite biology in ways that limit its relevance for modeling authentic developmental processes. As a result, we know little about how *T. gondii* adapts to, and is shaped by, the distinct physiological environments encountered in other host cell types [9, 10].

While transcriptional differences between parasites grown in distinct host cells have been reported [11], the accompanying epigenetic changes remain poorly characterized. In particular, it is unclear how host cell identity influences chromatin states that regulate gene expression over the course of infection. Astrocytes (ASTs), abundant in the central nervous system, represent a biologically relevant alternative host cell type that differs markedly from HFF in physiology and intracellular environment. Previous transcriptomic analyses from our group have shown that parasites grown in ASTs diverge in gene expression profiles from those in HFFs within days of infection, with differences in developmental markers and stage-specific transcription factors [12]. Comparing parasites in these two settings provides an opportunity to define how distinct host contexts shape the parasite’s epigenetic landscape and long-term developmental trajectory.

Understanding how *T. gondii* maintains transcriptional programs over extended periods in distinct host environments requires examining the chromatin states that persist beyond the initial stages of infection [13]. Epigenetic mechanisms, including histone variants and post-translational modifications (PTMs) such as methylation and acetylation, play central roles in regulating *T. gondii* gene expression during development [14]. These modifications can act as stable regulatory signals, shaping long-term gene expression patterns and contributing to parasite persistence [15]. Among these, histone 3 lysine 4 tri-methylation (H3K4me3) is associated with transcriptional activation, while histone 3 lysine 9 trimethylation (H3K9me3) is linked to gene silencing and heterochromatin formation [16]. In this work, we used developmentally competent *T. gondii* bradyzoites excysted from in vivo cysts to compare parasite adaptation in AST and HFF cells. We integrated ChIP-seq profiling of H3K4me3 and H3K9me3 with RNA-seq to examine how host cell identity shapes the parasite’s chronic chromatin landscape and transcriptional programs. Our results show that astrocyte and fibroblast environments impose distinct epigenetic states that coincide with divergent transcriptional trajectories and developmental outcomes, revealing a strong host cell influence on *T. gondii* long-term adaptation.

## Results

### Differences in parasite growth and developmental marker expression in astrocytes and fibroblasts

**Fig. 1:**
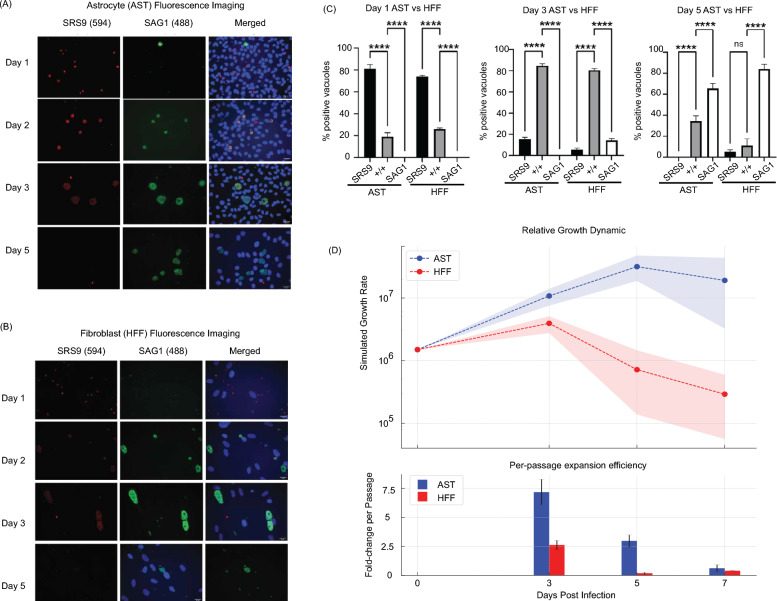
Bradyzoite-to-tachyzoite recrudescence differs between AST- and HFF- cultured parasites. **A**–**B** Immunofluorescence imaging of parasites undergoing recrudescence in ASTs (**A**) and HFFs (**B**). Cultures were harvested on the indicated days after infection and stained for the bradyzoite marker SRS9 (red), tachyzoite marker SAG1 (green), and host/parasite DNA (DAPI, blue). **C** Quantification of marker-positive vacuoles in AST and HFF at Days 1, 3, and 5. Bars represent the mean ± SD of triplicates. Statistical significance was assessed by Student’s *t*-test; ns = not significant. **D** Parasite population dynamics. D-top: Simulated cumulative parasite counts from Day 0–7, generated by propagating fold-change measurements across culture passages (median trajectory ± 95% interval, dashed lines with shaded bands). D-bottom: Per-passage expansion efficiency expressed as fold-change relative to input (mean ± SD, triplicates). Blue, AST; red, HFF

We previously developed a new ex vivo model of *Toxoplasma gondii* recrudescence using a Type II ME49 strain that has never been adapted to cell culture that preserved its ability to form abundant brain tissue cysts in mice [12]. In this model not only dormant bradyzoites convert into a fast-growing tachyzoite stage to then predictably shift into a slow-growing tachyzoite stage, a sequence that is independent of host immune pressure or host cell type. We also demonstrated that in astrocytes (but not fibroblasts), bradyzoites can also undergo bradyzoite-to-bradyzoite replication with cyst wall formation, revealing a host cell–dependent pathway that amplifies the cyst-forming population supporting the idea that excysted bradyzoites are multifunctional and developmentally plastic [12]. To confirm previous results, we inoculated freshly excysted Type II ME49 bradyzoites (ME49EW) into neonatal mouse astrocytes (ASTs) or human foreskin fibroblasts (HFFs); using the same ex vivo system to extend those findings under matched culture conditions. Parasite stage composition was monitored by immunofluorescence staining for the bradyzoite marker SRS9 and the tachyzoite marker SAG1 (Fig. 1A–B). In AST, vacuoles retained mixed SRS9⁺/SAG1⁺ expression through Day 5, consistent with a mixed or successfully propagating population. In contrast, HFF cultures progressively lost SRS9 expression, with SAG1⁺ tachyzoites predominating by Day 5. Further quantification confirmed these trends: AST maintained higher proportions of both SRS9⁺ and SAG1⁺ vacuoles, whereas HFF showed a marked reduction in any SRS9⁺ expression with an equivalent rise in SAG1-only populations (Fig. 1C).

Growth measurements aligned with these stage-specific differences. In AST, parasite numbers increased ~ sevenfold by Day 3, consistent with reawakening into early tachyzoites, and expanded nearly threefold between Days 3–5 in a rapid proliferative phase. By Day 7, expansion slowed but remained detectable, consistent with a transition toward a slower-replicating tachyzoite state. By contrast, HFF cultures expanded only ~ 2–threefold by Day 3 and showed no further increase, thereafter, reflected by reduced expansion efficiency (< onefold change). Simulated cumulative trajectories, with 95% confidence intervals, mirrored these patterns (Fig. 1D), indicating that AST-cultured parasites sustain sequential rounds of proliferation with eventual transition into a slower-replicating tachyzoite state, while fibroblasts support only a transient burst of tachyzoite growth before failing to maintain replication.

Together, these results reaffirm and expand on previously reported findings [12] that AST hosts provide a permissive environment for prolonged parasite expansion and maintenance of bradyzoite traits, whereas fibroblasts favor a short-lived burst of tachyzoite growth followed by decline. These observations confirmed fundamental differences in host-dependent parasite persistence and provide the experimental foundation for the chromatin and transcriptomic analyses that follow.

### Global differences in promoter H3K4me3 landscapes between host environments

**Fig. 2:**
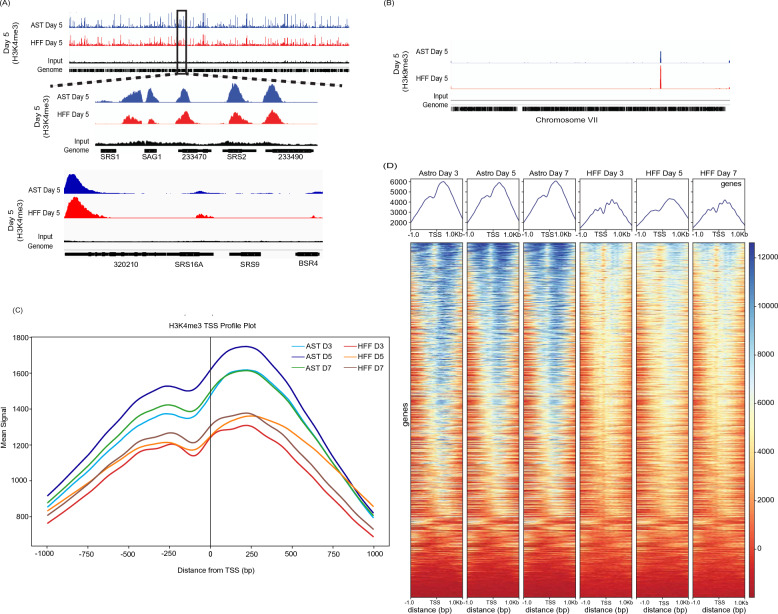
Global profiles of histone modifications in AST- and HFF-cultured parasites. **A** Genome browser snapshots showing H3K4me3 enrichment at representative promoters on Day 5. H3K4me3 peaks demonstrate its typical role in accumulating at the promoters of several genes on Chromosome VII, including the tachyzoite marker SAG1 with other nearby genes and the bradyzoite marker SRS9. Parasites cultured in astrocytes (AST, blue) and in fibroblasts (HFF, red) are shown. Input is indicated in black. **B** Representative locus on Chromosome VII highlighting H3K9me3 enrichment at centromeric heterochromatin on Day 5. Both AST- and HFF-cultured parasites exhibit strong focal H3K9me3 peaks, consistent with typical heterochromatin localization at centromeres and telomeric regions. **C** Metagene profiles of H3K4me3 enrichment across the TSS of all annotated genes (-1 to + 1 kb). AST-cultured parasites display stronger and broader promoter enrichment compared to HFF-cultured parasites at all time points. **D** Heatmaps of H3K4me3 signal at the TSS ± 1 kb for a curated set of 1700 cell cycle–related genes across Day 3, Day 5, and Day 7. The profile plots reveal persistent promoter-proximal enrichment in AST parasites, whereas HFF parasites show decreased H3K4me3 occupancy

Because transcriptional divergence alone cannot explain the long-term developmental differences between host cell types, we next examined histone-modification profiles to determine whether chromatin state contributes to these host-dependent outcomes. To investigate how host cell context shapes the *T. gondii* chromatin landscape, we profiled two histone modifications with contrasting roles: the activating mark H3K4me3 and the repressive, heterochromatin-associated mark H3K9me3. ChIP–seq was performed on parasites cultured in ASTs or HFF collected at Days 3, 5, and 7 post-infection as described above. Replicate correlations were high for both marks (H3K4me3: r > 0.97 in AST, r > 0.96 in HFF; H3K9me3: r > 0.95 in both), confirming reproducibility and revealing clear clustering by host cell type (Supplementary Fig. 1). Sequencing depth, alignment rates, and duplicate removal statistics for all ChIP-seq libraries are summarized in Supplementary Table S1. Genome-browser views at representative promoters (Fig. 2A) highlight robust H3K4me3 peaks in both conditions, consistent with its classical association with transcriptional activity. In contrast, H3K9me3 tracks at Day 5 (Fig. 2B) showed sharp enrichment at centromeric and telomeric regions in both environments, consistent with canonical heterochromatin localization and largely independent of host environment, indicating that parasites in both astrocytes and fibroblasts maintain normal heterochromatin organization and growth behavior. To evaluate whether additional active histone marks mirrored H3K4me3 enrichment patterns, we performed exploratory profiling of H3K9ac and H3K14ac. Promoter-proximal signals for H3K9ac were strongly correlated with H3K4me3 across genes, TSS-centered profiles and gene level analysis through IGV demonstrated similar promoter-centered enrichment patterns (Supplementary Figure S2A–C). H3K14ac exhibited broader enrichment with increased variability between biological replicates, limiting its suitability for consistent quantitative comparisons. These results support the use of H3K4me3 as the primary active chromatin mark for downstream analyses.

Genome-wide transcription start site (TSS) profiles of H3K4me3 (± 1 kb) revealed consistent differences between host environments (Fig. 2C). AST-cultured parasites displayed stronger and broader promoter enrichment, with peak densities approximately 1.3-fold higher than in HFF. This elevated signal in AST suggests a more transcriptionally engaged and permissive chromatin state relative to fibroblast-grown parasites. To test whether this pattern extended to functionally important loci, we examined ~ 1700 genes annotated for cell-cycle and replication-related processes, representing a functionally defined subset of promoters linked to growth and division. TSS-centered heatmaps for this subset (Fig. 2D) showed elevated and more uniform H3K4me3 enrichment in AST-cultured parasites compared with their fibroblast counterparts. To determine whether stage-marker expression differences were accompanied by promoter-level chromatin changes, we quantified H3K4me3 enrichment at the SAG1 and SRS9 loci using TSS-centered DeepTools matrices (± 1 kb). SAG1 maintained strong promoter-associated H3K4me3 across host environments, whereas SRS9 exhibited low and largely unchanged promoter marking in both conditions (Supplementary Figure S3). Together, these results indicate that the astrocyte environment supports promoter accessibility at genes critical for replication and division, whereas the fibroblast environment is associated with a comparatively globally diminished promoter marking.

### Temporal dynamics of H3K4me3 enrichment differ between astrocyte and fibroblast cultures

**Fig. 3:**
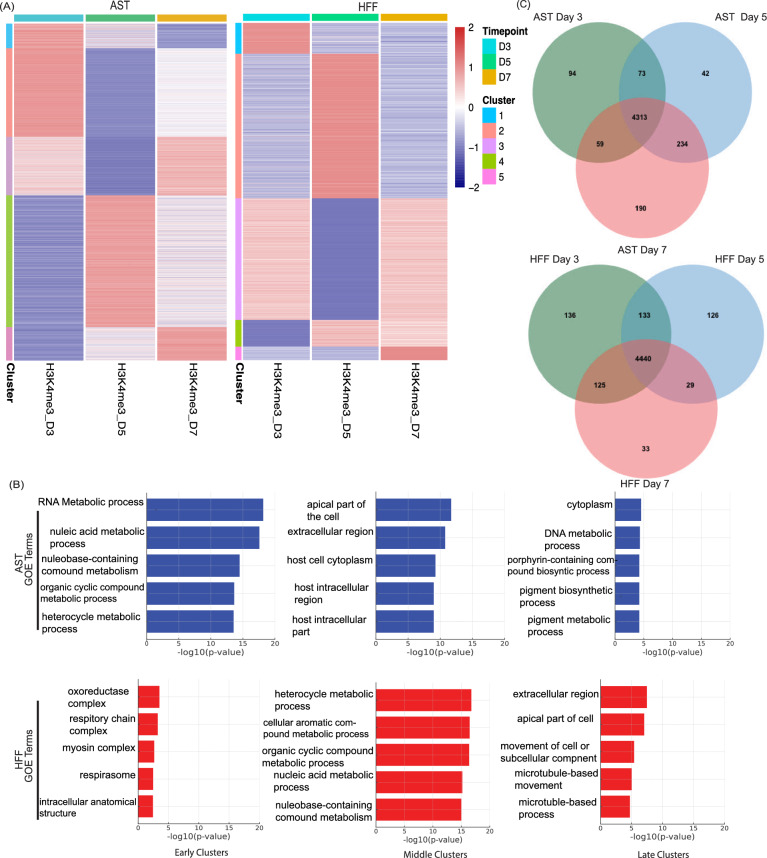
Divergent temporal dynamics of H3K4me3 enrichment in AST- versus HFF-cultured parasites. **A** Heatmaps of H3K4me3 signal at the TSS ± 1 kb for all genes, z-scored per gene. Genes were grouped by k-means clustering (k = 5). ASTs exhibit a broad early wave of promoter H3K4me3 at D3 and shifts to a replication and differentiation program at D7, whereas HFFs show limited marking at D3 with expansion at D5–D7. **B** GO over-representation analysis for early, mid, and late H3K4me3 clusters in each condition (terms ranked by significance). **C** Venn diagrams showing overlap of genes with significant H3K4me3 peaks at each time point within AST and HFF

Having established global differences in promoter H3K4me3, we next examined temporal changes across Days 3, 5, and 7 (Fig. 3A), important time points for diverging patterns of parasite growth. Complete H3K4me3 peak lists for each condition and time point are provided in Supplementary Table S2. Clustering of promoter-associated H3K4me3 (z-scored per gene) revealed coordinated programs that differed depending on host environment. In ASTs, a broad early wave of enrichment was evident at Day 3, with GO categories dominated by RNA and nucleotide metabolism together with apical/organelle-associated terms. Day 5 was enriched for transcriptional and chromatin-related functions, and by Day 7, enrichment shifted toward cytoskeletal and motility processes. In HFFs, promoter marking was limited at Day 3, with modest enrichment for metabolic categories (e.g., oxidoreductase and vesicle/energy-related terms). By Days 5–7, HFFs showed a marked expansion of H3K4me3, with middle and late clusters enriched for mitochondrial respiration, proteostasis, and microtubule-based pathways (Fig. 3B). Full GO enrichment results for the H3K4me3 clusters are provided in Supplementary Table S3**.**

Peak-overlap analysis indicated that most H3K4me3-marked promoters were retained across time, with additional day-specific gains and losses in both conditions (Fig. 3C). In astrocyte cultures, 4,313 promoters were shared across all three timepoints, while 326 were detected at only a single day. Fibroblast-grown parasites similarly retained the majority of marked promoters across time, with 4,440 shared across all three days and 292 detected at a single timepoint. However, comparing AST- and HFF-grown parasites at matched timepoints revealed that divergence in promoter-associated H3K4me3 was driven primarily by differential enrichment at shared genomic loci rather than by condition-specific peak acquisition. This effect was most pronounced at Day 5, when the majority of overlapping promoters showed substantial differences in H3K4me3 enrichment between host environments. Consistent with our cluster analysis, many programs showing astrocyte-biased enrichment at this time point corresponded to transcriptional regulators and genes linked to cell-cycle progression and developmental competence, including most AP2 transcription factors, such as AP2XI-4 and AP2XII-1. In contrast, HFF-grown parasites did not exhibit comparable differential enrichment at regulatory transcription factors, instead showing H3K4me3 changes distributed across metabolic, proteostatic, and cytoskeletal pathways. Altogether, these data indicate that parasite chromatin states diverge early between astrocyte and fibroblast cultures, leading to sustained differences in metabolism, growth potential, and developmental competence.

### Transcriptomic differences between host cell environments

**Fig. 4:**
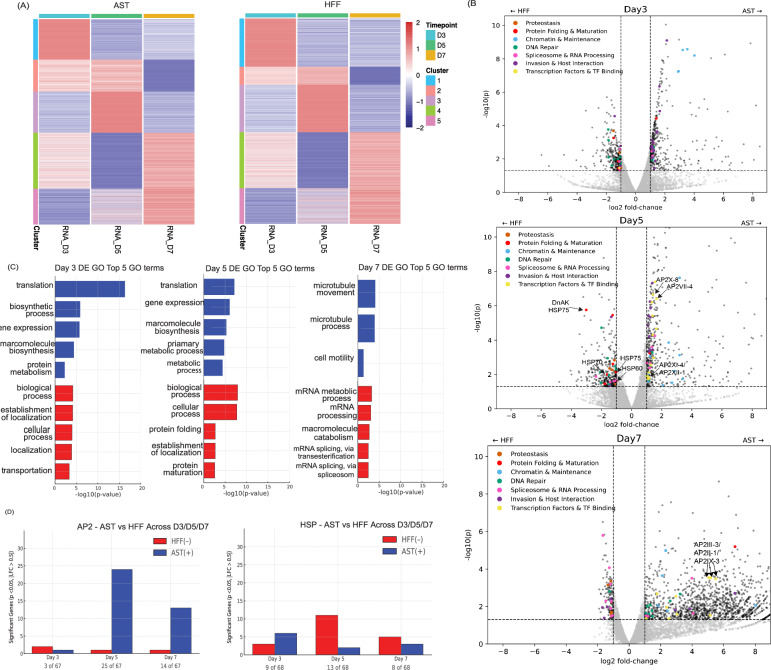
Transcriptomic differences between AST- and HFF-cultured parasites across time. **A** Heatmaps of RNA-seq expression (z-scored per gene). Genes were grouped by k-means clustering (k = 5) and displayed across Day 3, Day 5, and Day 7 in AST and HFF. AST-cultured parasites show broad transcriptional activation at early time points, whereas HFF-cultured parasites exhibit delayed but extensive reprogramming at later stages. **B** Volcano plots of differentially expressed genes between conditions at each day (raw p < 0.05; |log₂FC|≥ 1). Some notable H3K4me3 marked gene groups include: proteostasis, protein folding and maturation, RNA processing and spliceosome components, Apical and host interaction or mobility, genome maintenance, and transcription factors. **C** The top five enriched GO terms at each time point for both AST (blue) and HFF (red). **D** Differential expression of transcription factor (AP2) and chaperone (HSP) families across conditions. AST cultures are enriched for AP2 transcription factors, consistent with regulatory remodeling. HFF cultures show greater induction of HSP/chaperone genes, consistent with stress-associated proteostasis. Bars indicate the number of genes significantly differentially expressed (p-value < 0.05; |log₂FC|≥ 0.5)

To confirm whether epigenomic differences were reflected at the transcript level, we performed RNA-seq in parasites grown in ASTs or HFFs collected at days 3, 5, and 7. Global expression profiles diverged over time (Fig. 4A). Replicate correlations were high and samples clustered primarily by host cell type, confirming reproducibility of the RNA-seq datasets (Supplementary Figure S4). As expected, there were several differentially expressed (DE) genes separated the two conditions (padj < 0.05): Day 3—235 (185 AST-enriched, 50 HFF-enriched), Day 5—541 (433 AST, 108 HFF), and Day 7—349 (290 AST, 59 HFF). Volcano plots (Fig. 4B) highlight these shifts, with broader distributions emerging by Days 5–7. Full DE results for RNA-seq are provided in Supplementary Table S4**.**

GO analysis of DE genes revealed distinct temporal emphases (Fig. 4C), and the complete list can be found in Supplemental Table S5. At Day 3, parasites grown in ASTs were enriched for translation and gene expression terms (ribosome, peptide biosynthesis, and RNP assembly), whereas HFFs showed comparatively limited enrichment. By Day 5, ASTs added RNA-metabolic and transcription-related functions, including transcription factor activity with several notable AP2 entries, while HFFs instead showed prominent enrichment for strong core spliceosomal machinery as well as protein folding and chaperone-mediated proteostasis. At Day 7, ASTs shifted toward cytoskeletal and motility-associated terms (microtubule-based movement, motor activity), whereas HFFs continued to emphasize RNA processing, spliceosome components, and proteostasis pathways.

Gene family analyses underscored distinct regulatory trajectories in each host environment (Fig. 4D, the full list of which are provided in Supplementary Table S6. In ASTs, AP2 transcription factors showed their strongest induction at Day 5, consistent with the enrichment of transcriptional and chromatin-related functions. Notably, AP2XI-4, a regulator of bradyzoite gene expression required for proper cyst formation, and AP2XII-1, which governs endodyogeny and growth, were among the induced factors. Both are central to developmental control and their temporal induction points to a transcriptionally active and complex phase in AST-cultured parasites. A smaller subset of AP2s persisted into Day 7 alongside cytoskeletal and invasion-associated genes. In contrast, HSP/chaperone genes were most strongly induced at Day 5 in HFF, aligning with the proteostasis and protein folding terms highlighted by GO analysis. Prominent examples included HSP60, HSP70, and the HSP70-interacting protein HIP70, with expression remaining elevated at Day 7. This chaperone program paralleled the late enrichment of spliceosome and proteasome components, suggesting a stress-associated shift toward RNA and protein quality control in fibroblast cultures. By Day 5, transcriptomic differences between ASTs and HFFs paralleled the divergence already evident in promoter H3K4me3 profiles, indicating that each dataset captures a significant shift in regulatory programming and encouraged a combined analysis of chromatin and transcriptional dynamics.

### Integrated chromatin–transcriptome dynamics

**Fig. 5:**
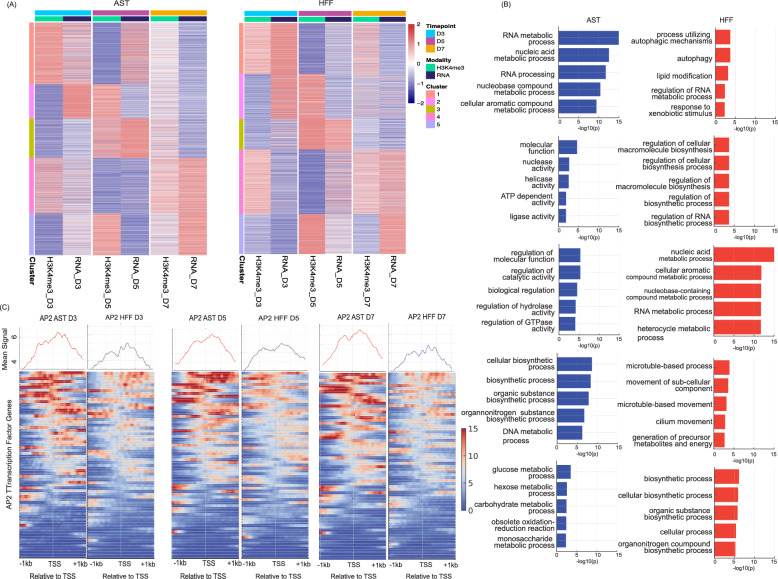
Integrated chromatin–transcriptome programs across time in AST- and HFF-cultured parasites. **A** Paired heatmaps of H3K4me3 and RNA for all genes at Days 3, 5, and 7 in AST and HFF (z-scored per gene within data type; columns in progressing time). Rows are grouped by k-means (k = 5) to reveal coordinated chromatin–RNA waves. **B** Top five GO terms per cluster. **C** Profile plots over AP2 transcription start sites showing elevated proximal-promoter H3K4me3 in ASTs compared to HFFs

To determine how promoter activation and transcriptional changes are coordinated across time and host environments, we integrated H3K4me3 and transcriptomic data across Days 3, 5, and 7 in AST and HFF, grouping genes into five clusters (Fig. 5A). The paired heatmaps and BP enrichments (Fig. 5B) mirror the single-modality results but resolve their joint or divergent timing as coordinated epigenomic and transcriptomic programs.

In ASTs, two Day-3–dominant clusters are evident. The first shows concordant elevation of H3K4me3 and RNA at Day 3, with GO categories enriched for RNA and nucleic-acid metabolism (ribosome biogenesis, peptide biosynthesis, and RNA processing), consistent with rapid engagement of core gene-expression machinery. A second RNA-first cluster exhibits elevated transcripts at Day 3 with promoter H3K4me3 strengthening by Day 5. Given its enrichment for chromatin organization, DNA metabolism, and ATP-dependent helicase/remodeling factors, this pattern is consistent with early induction of chromatin remodelers, whose promoter marks consolidate as the epigenomic profile changes. The program then pivots to a discrete Day-5 block (Cluster 3) in which H3K4me3 and RNA peak together and GO terms emphasize transcription/chromatin regulation and catalytic/GTPase activity, consistent with a mid-course regulatory pulse. By Day 7, AST parasites shift into late-stage execution modules, corresponding to Clusters 4 and 5, which show coordinated increases in both H3K4me3 and RNA abundance. These clusters are enriched for cytoskeletal and motility-associated programs (e.g., microtubule-based movement and motor activity), and their selective activation at Day 7 is clearly resolved in Fig. 5B, where the heatmaps display their distinct late-peaking H3K4me3 and transcript signatures.

In HFFs, promoter marking and RNA changes are muted at Day 3, yet enrichment already includes autophagy, xenobiotic response, lipid modification, and RNA-related categories, indicating an early response distinct from the gene-expression–dominated AST program. By Days 5–7, HFFs exhibit a broader induction: clusters expand in both assays with GO terms spanning metabolic pathways, substantial representation of proteostasis and chaperone functions, and RNA processing and spliceosome components. Relative to ASTs, HFFs show a delayed and different trajectory for its epigenomic remodeling, prioritizing protein and RNA quality-control (Fig. 5B).

To test whether promoter marking precedes and spans transcriptional changes, we examined AP2 transcription factor loci (Fig. 5C). In ASTs, promoter-proximal H3K4me3 was already elevated at Day 3 (1.2 times higher than HFFs) and remained high through Day 7; AP2 transcripts rose at Day 5, indicating that promoter marking preceded the transcriptional wave and persisted over time. In HFFs, AP2 promoters showed lower H3K4me3 enrichment across time with correspondingly weak/delayed transcript induction. Thus, at regulatory genes, promoter H3K4me3 in ASTs both precedes and accompanies transcriptional activation, whereas in HFFs the lack of H3K4me3 enrichment aligns with limited AP2 up-regulation and a shift toward later proteostasis/processing programs. These divergent trajectories highlight how the host cell environment shapes the timing and character of coordinated chromatin–RNA programs.

## Discussion

The host cell environment is increasingly recognized as a critical determinant of *Toxoplasma gondii* developmental biology [17]. While fibroblasts have long served as the standard in vitro model, they do not recapitulate the diverse tissue niches encountered during infection, particularly within the central nervous system where chronic persistence is established [18]. Understanding how different host cells shape parasite gene regulation is therefore essential for interpreting in vitro studies and for uncovering the mechanisms that enable long-term survival in vivo.

Using combined epigenomic and transcriptomic profiling, we demonstrated that the host cell environment plays a pivotal role in modulating promoter marking and transcriptional adaptability in *Toxoplasma gondii*. Parasites cultured in ASTs exhibited global elevation of H3K4me3 enrichment, consistent with an open promoter state and activation of gene programs involved in replication and differentiation [19, 20]. In contrast, parasites grown in HFFs showed reduced H3K4me3 levels, suggestive of a more restrictive promoter landscape that biases the parasite toward transcriptional repression, stress adaptation, and reduced developmental plasticity [21]. H3K9me3 remained sharply localized at centromeres and select telomeric regions and was largely invariant across host environments, indicating that constitutive heterochromatin domains are unlikely to drive the observed differences. These chromatin-level distinctions mirror the phenotypic outcomes we observed, where AST-grown parasites sustained sequential rounds of proliferation with retention of bradyzoite-associated traits, whereas fibroblast-grown parasites supported only a transient tachyzoite burst followed by collapse. Consistent with this interpretation, promoter-proximal H3K4me3 at the stage-marker loci SAG1 and SRS9 did not simply track marker positivity, indicating that host-dependent chromatin differences extend beyond stage-marker expression alone (Supplementary Figure S3).

Epigenetic re-programming is a conserved feature among eukaryotes, enabling parasites to modulate gene expression in response to environmental conditions [22, 23]. This adaptability was particularly evident in ASTs, where H3K4me3 deposition at AP2 transcription factor loci was already elevated at Day 3 and preceded transcript induction by Day 5. Notably, the induced set encompassed a broad cohort of ApiAP2s**,** including AP2XI-4**,** AP2XII-1**,** AP2XII-4/5**,** AP2VIIa-3/5**,** AP2VIII-4/6**,** and AP2X-9**,** associated with developmental regulation, replication control, and chromatin remodeling [24–27]. Within this group, AP2XII-8 was previously identified as a ribosome-regulon driver [22], and links transcriptional activation to translational regulation. Together, these data reveal a multifactorial AP2 cascade underlying the coordinated mid-course regulatory pulse observed in astrocyte-grown parasites. By Day 7, AST parasites transitioned to cytoskeletal and motility-associated programs, consistent with remodeling into a slower-replicating, motile tachyzoite state [28].

In contrast, HFFs displayed a distinct and delayed trajectory. Promoter marking at Day 3 was muted but already associated with autophagy and xenobiotic-response terms, suggesting early stress sensing rather than broad transcriptional engagement [29]. By Day 5, HFF parasites showed strong induction of proteostasis and chaperone pathways, including DnaJ (HSP40s), HSP60, HSP70, and the HSP70-interacting protein HIP70, coupled with proteasome- and spliceosome-associated terms that remained prominent through Day 7. These findings point to a fibroblast environment that imposes constraints and triggers stress-associated remodeling programs, rather than supporting broad transcriptional plasticity. Similar host-imposed metabolic and proteostatic pressures have been described in other intracellular pathogens, where adaptation is shaped more by survival under stress than by developmental progression [7, 30, 31].

Together, these results establish that host-specific chromatin states underlie divergent developmental trajectories [32]. These trajectories align with passage-wise growth outcomes (Fig. 1D), with ASTs maintaining expansion beyond Day 3 and HFF failing to do so. AST-cultured parasites engage early gene expression and regulatory programs that enable developmental flexibility, whereas HFF-cultured parasites are driven into stress-centered responses that fail to sustain long-term replication. These observations emphasize that the choice of model significantly influences interpretations of *T. gondii* biology. Fibroblast-based systems, while widely used, may underestimate the parasite’s transcriptional and epigenetic plasticity by biasing toward stress-adaptation pathways [33, 34]. Astrocyte-based models may better approximate the CNS environment where chronic infection is established, providing a framework to link chromatin–transcriptional dynamics with parasite persistence and to more faithfully model infection for future interventions.

## Methods

### Maintenance of parasite and host cells

Lab-adapted ME49 strains of *Toxoplasma gondii* were propagated in high-glucose Dulbecco’s Modified Eagle Medium (DMEM) supplemented with 5% heat-inactivated fetal bovine serum (FBS). Human foreskin fibroblasts (HFFs) were cultured in DMEM containing 10% (v/v) heat-inactivated fetal bovine serum (FBS), supplemented with 100 µg/mL streptomycin, 100 U/mL penicillin, and 10 µg/mL gentamycin. Both host cells and parasites were maintained in a humidified incubator at 37 °C with 5% CO₂ and 21% O₂.

### Maintenance of ex vivo bradyzoites

Developmentally competent ME49EW bradyzoites were cultured on HFFs or astrocytes in a hypoxic chamber. Cultures were maintained in DMEM supplemented with 5% (v/v) FBS**,** 2 mM L-glutamine**,** 1 mM sodium pyruvate**,** and antibiotics (100 µg/mL streptomycin, 100 U/mL penicillin, and 10 µg/mL gentamycin), buffered with 25 mM HEPES. The hypoxic conditions were achieved using a gaseous mixture of 90% nitrogen, 5% oxygen, and 5% CO₂ at 37 °C.

### Murine astrocyte culture

Primary astrocytes were isolated from the brain cortices of newborn (0–3 days old) C57BL/6 mouse pups. Brain tissue was homogenized in ice-cold isolation media (DMEM with 2% heat-inactivated FBS) and filtered through a 40 µm mesh strainer. Cells were centrifuged at 2000 rpm for 10 min at 4 °C, resuspended in washing media, and centrifuged again. The final cell pellet was resuspended in pre-warmed astrocyte media (composition: DMEM supplemented with 10% FBS**,** 1% non-essential amino acids (NEAA)**,** 1% GlutaMAX**,** 100 U/mL penicillin and 100 µg/mL streptomycin**,** and 10 mM HEPES**),** mixed, and seeded into non-vented T25 cm^2^ flasks. Cultures were maintained at 37 °C with 5% CO₂, and media were refreshed on day 4. On day 10, non-adherent cells were dislodged by shaking the flask at 260 rpm for 2 h at 37 °C, followed by overnight shaking at 100 rpm. Astrocytes were detached using 0.25% Trypsin–EDTA, reseeded into T75 flasks at a density of 1 × 10⁶ cells per flask, and used for recrudescence experiments upon reaching confluence.

### Immunofluorescence assays

Parasite growth and stage-marker expression were assessed by immunofluorescence at the indicated time points post-infection. Infected AST and HFF monolayers were fixed in 4% paraformaldehyde, permeabilized with acetone, and blocked in 5% donkey serum in PBS. Samples were incubated overnight at 4 °C with rabbit anti-SRS9 (1:1000) and mouse anti-SAG1 (1:1000; Bio-Rad, cat. no. 9070-2020). Where indicated, biotinylated Dolichos biflorus agglutinin (DBA; Vector Laboratories, cat. no. B-1035-5; 1:500) was used to visualize cyst wall formation. After washing, cells were incubated for 1 h at room temperature with donkey anti-rabbit Alexa Fluor 568 (Invitrogen, cat. no. A10042), goat anti-mouse Alexa Fluor 488 (Invitrogen, cat. no. A-11029), and streptavidin-Alexa Fluor 647 (Sigma-Aldrich, cat. no. S32357), all at 1:1000 dilution. Coverslips were mounted with DAPI-containing antifade medium (Vectashield Vibrance, Vector Laboratories, cat. no. H-1800). Images were acquired using a Leica DMI6000 fluorescence microscope with a 40 × objective, and vacuoles were scored manually across at least three independent biological replicates per condition.

### Chromatin immunoprecipitation sequencing (ChIP-seq)

#### ChIP preparation

Approximately 20 million Type II ME49 parasites were pelleted and crosslinked with 1% formaldehyde for 10 min at room temperature, then quenched with 0.125 M glycine. The crosslinked pellets were washed twice with phosphate-buffered saline (PBS) and resuspended in 1 mL of nuclear extraction buffer (10 mM HEPES, 10 mM KCl, 0.1 mM EDTA, 0.1 mM EGTA, 1 mM DTT, 0.5 mM AEBSF, 1X Roche protease inhibitor, and 1X Roche phosphatase inhibitor). After a 30-min incubation on ice, 10% Igepal CA-630 was added, and the samples were homogenized by passing through a 26G × ½ needle. Nuclear pellets were obtained by centrifugation at 5,000 rpm and resuspended in shearing buffer (0.1% SDS, 1 mM EDTA, 10 mM Tris–HCl pH 7.5, and 1X Roche protease/phosphatase inhibitors). Chromatin was fragmented using a Covaris S220 sonicator (5 min, duty cycle 5%, intensity 140 W, 200 cycles/burst, 6 °C).

Equal volumes of ChIP dilution buffer (30 mM Tris–HCl pH 8, 3 mM EDTA, 0.1% SDS, 30 mM NaCl, 1.8% Triton X-100, and 1X Roche inhibitors) were added to the sonicated chromatin. The samples were pre-cleared with 13 μL of protein A agarose/salmon sperm DNA beads washed in ChIP dilution buffer without inhibitors. After 1 h of agitation at 4 °C, ~ 10% of each sample was set aside as input DNA, and the remaining chromatin was incubated with 2 μL of anti-H3k4me3, anti-H3k9me3 antibody (Abcam ab8580 and ab8898), anti-H3K9ac and anti-H3k14ac (Diagenod C15210015 and C15410310) overnight at 4 °C. Immune complexes were captured using protein A agarose/salmon sperm DNA beads blocked with 1 mg/mL BSA. Beads were washed sequentially (twice with low salt buffer, twice with high salt buffer, twice with LiCl buffer, and twice with TE buffer). DNA–protein complexes were eluted with 1% SDS/0.1 M sodium bicarbonate buffer. Crosslinks were reversed overnight at 45 °C with 55 μL of 5 M NaCl.

Samples were treated with 15 μL of RNAse A (20 mg/mL) at 37 °C and 2 μL of proteinase K (20 mg/mL) at 45 °C. DNA was extracted using phenol/chloroform, precipitated with ethanol, and purified with AMPure XP beads. Libraries were prepared using the KAPA HyperPrep kit (KK8504) and sequenced on the Illumina NextSeq 500 platform.

#### ChIP-seq data analysis

Read quality was assessed using FastQC (v0.11.8) [35]. Adapter sequences and low-quality bases were trimmed using Trimmomatic (v0.39) [36] and Sickle (v1.33) [37]Reads were aligned to the *Toxoplasma gondii* ME49 (v62) genome assembly using Bowtie2 (v2.4.4) [38], and duplicate reads were removed with Picard Toolkit [39]. Properly paired reads with mapping quality ≥ 40 were retained using SAMtools (v1.11) [40]. Genome coverage was calculated and normalized to reads-per-million using BEDTools (v2.27.1) [41]. Signal profiles and correlation plots were generated with deepTools2 (v2.5.7) [42] and browser tracks were visualized in the Integrative Genomics Viewer (IGV) [43]. Heatmaps and metagene analyses were generated in R to compare H3K4me3 and H3K9me3 enrichment profiles.

### RNA-seq processing for gene expression analysis

FASTQ files were evaluated for quality using FastQC [35]. Adapter trimming and removal of the first 11 bp of each read were performed with Trimmomatic (v0.39) [36], and low-quality reads (Phred < 25) were discarded with Sickle (v1.33) [37]. Reads were aligned to the *T. gondii* ME49 genome (V62) using HISAT2 (v2.2.1) [44], and properly paired reads with mapping quality ≥ 40 were retained using SAMtools [40]. Transcript assembly and expression quantification were performed with StringTie (v2.2.1). Normalized transcript abundance was expressed as TPM, and differential expression analyses were performed in DESeq2 [45].

## Supplementary Information

Additional file 1: Figure S1. Pearson correlation heatmap of H3K4me3 ChIP-seq profiles across host conditions. Pairwise Pearson correlations were computed from normalized read counts using the DiffBind package. Replicates from each condition are highly similarity (r > 0.8) with clear segregation between human foreskin fibroblast (HFF) and astrocyte (AST) infections.

Additional file 2: Figure S2. Genome-wide relationship between H3K4me3 and histone acetylation marks. (A) Spearman correlation analysis of promoter-proximal signal (±1 kb from annotated TSS) demonstrates strong concordance between H3K4me3, H3K9ac, and H3K14ac across genes. Correlation coefficients (ρ = 0.76–0.86) indicate substantial co-enrichment of active histone marks. (B) Aggregate TSS-centered profiles (±1 kb) show promoter-associated enrichment of H3K4me3 in both astrocytes (AST) and fibroblasts (HFF). H3K9ac and H3K14ac display similar promoter-centered distributions, consistent with active chromatin architecture. (C) Representative genome browser view illustrating co-localization of H3K4me3 and acetylation marks at actively transcribed loci, including SAG1. These results indicate that H3K9ac and H3K14ac broadly mirror H3K4me3 promoter enrichment patterns, supporting the use of H3K4me3 as the primary active chromatin mark for downstream analyses.

Additional file 3: Figure S3. Promoter-associated H3K4me3 signal at SAG1 and SRS9 loci. TSS-centered H3K4me3 profiles (±1 kb) were extracted from deepTools matrices for SAG1 (TGME49_233460) and SRS9 (TGME49_320190) in astrocytes (AST) and fibroblasts (HFF) at Day 3 and Day 5. Solid lines represent SAG1 and dashed lines represent SRS9. SAG1 shows strong promoter-proximal H3K4me3 enrichment in both host environments, whereas SRS9 remains near baseline without host-dependent gain of promoter-associated H3K4me3.

Additional file 4: Figure S4. Pearson correlation heatmap of RNA-seq expression profiles across host conditions. Pairwise Pearson correlations for library size normalized read counts. All replicates exhibited strong agreement (r > 0.9), confirming high sequencing reproducibility. Samples clustered primarily by host cell type (AST vs HFF).

Additional file 5: Table S1. Mapping statistics for ChIP-seq libraries. Summary of sequencing and alignment metrics for all ChIP-seq libraries generated in this study across host environments and time points. Reported values include total sequencing reads, uniquely aligned reads, and deduplicated read counts used for downstream analysis.

Additional file 6: Table S2. Complete H3K4me3 peak lists. All significant H3K4me3 peaks identified by MACS2 for AST and HFF parasites at Days 3, 5, and 7. Provided as separate broadPeak files packaged in a ZIP archive.

Additional file 7: Table S3. GO enrichment analysis for H3K4me3 clusters. Biological process, molecular function, and cellular component terms for each temporal H3K4me3 cluster.

Additional file8: Table S4. RNA-seq differential expression results (AST vs HFF). DESeq2 output tables for Days 3, 5, and 7, including log₂FC, adjusted p-values, and normalized expression.

Additional file 9: Table S5. GO enrichment results for RNA-seq datasets. Enrichment results for significantly up- or down-regulated gene sets at each time point.

Additional file 10: Table S6. Curated functional gene sets used in this study. Includes proteostasis/folding sets, AP2 transcription factors, chromatin regulators, and invasion/host-interaction gene families.

## Data Availability

All data are present in the main text and the supplementary materials. All biological materials and data are available from the authors upon request. ChIP-seq and RNAseq data are available in NCBI SRA https://www.ncbi.nlm.nih.gov/bioproject/PRJNA1433593 (PRJNA1433593).
