# Supplementary figures and images for: Host-cell dependent epigenetic profiles associated with survival outcomes in T. gondii infection
Source: Epigenetics Chromatin. 2026 May 5;19:27. doi: 10.1186/s13072-026-00678-x (PMC13317167; doi:10.1186/s13072-026-00678-x)

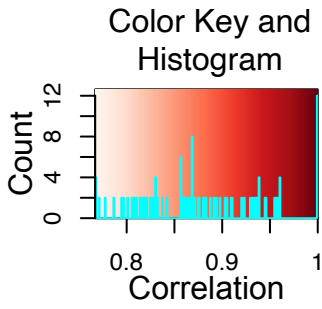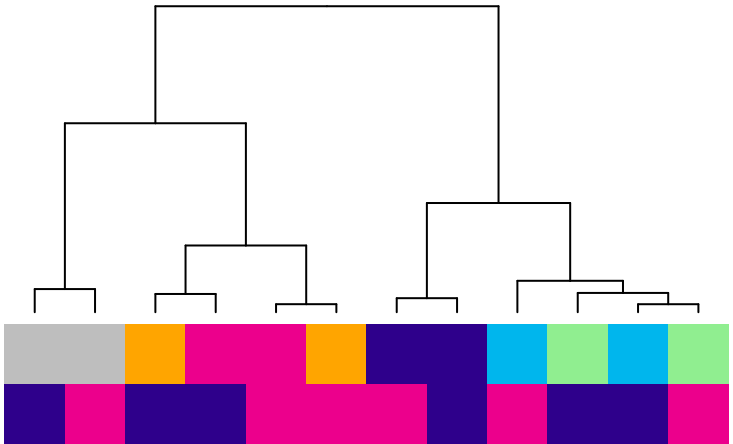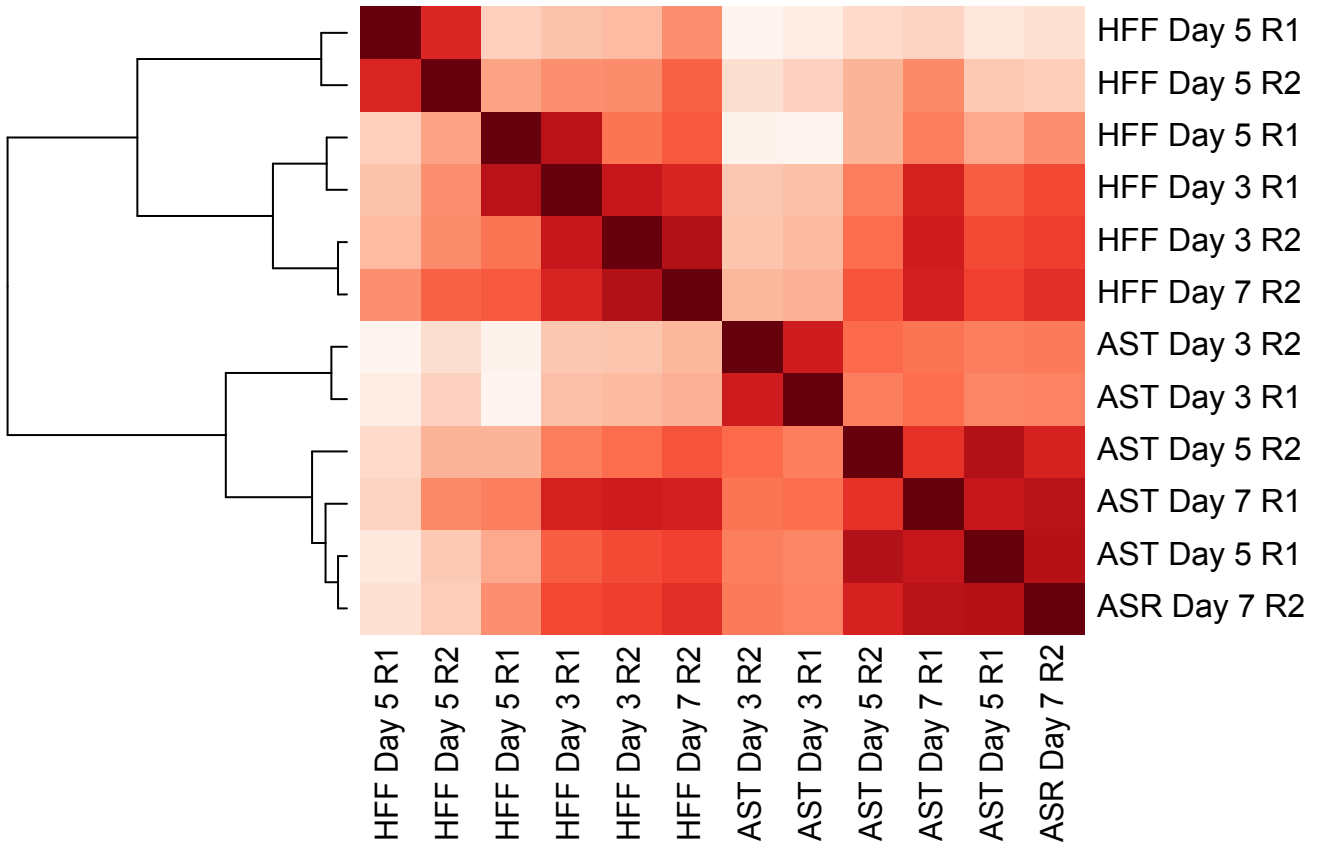

Supplement: Supplementary file 1 — Additional file 1: Figure S1. Pearson correlation heatmap of H3K4me3 ChIP-seq profiles across host conditions. Pairwise Pearson correlations were computed from normalized read counts using the DiffBind package. Replicates from each condition are highly similarity (r > 0.8) with clear segregation between human foreskin fibroblast (HFF) and astrocyte (AST) infections. [file 13072_2026_678_MOESM1_ESM.pdf]

(A) Spearman Correlations - H3K4me3, H3K9ac, H3K14ac

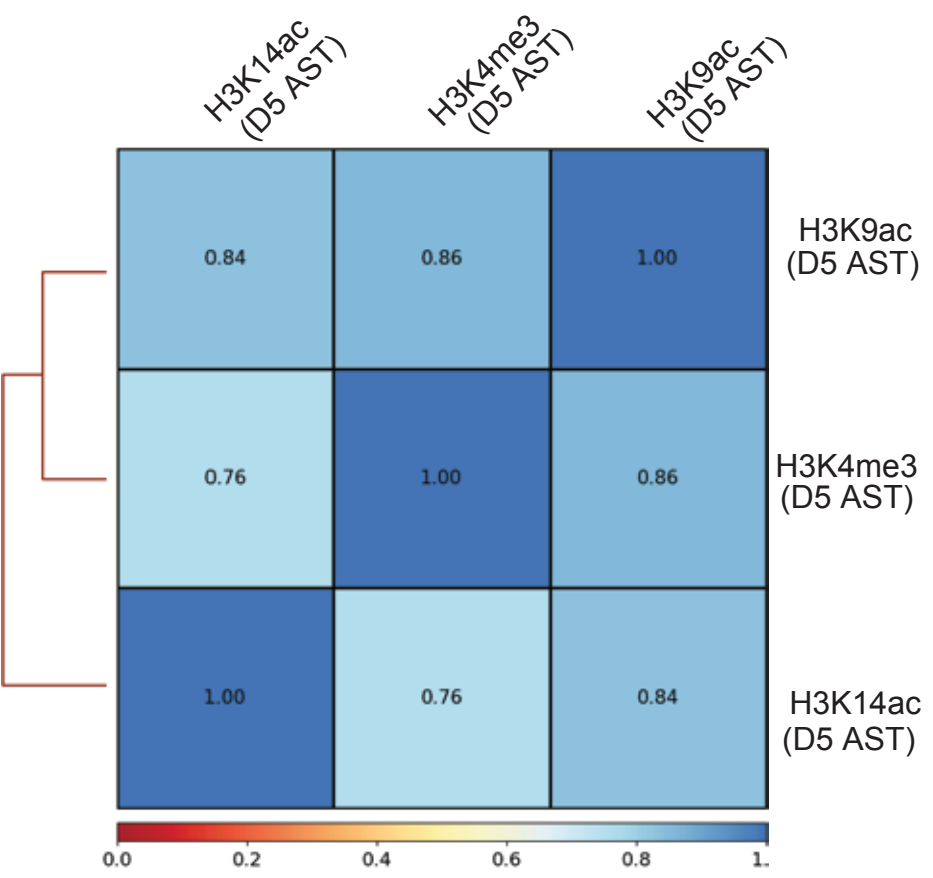

(C)

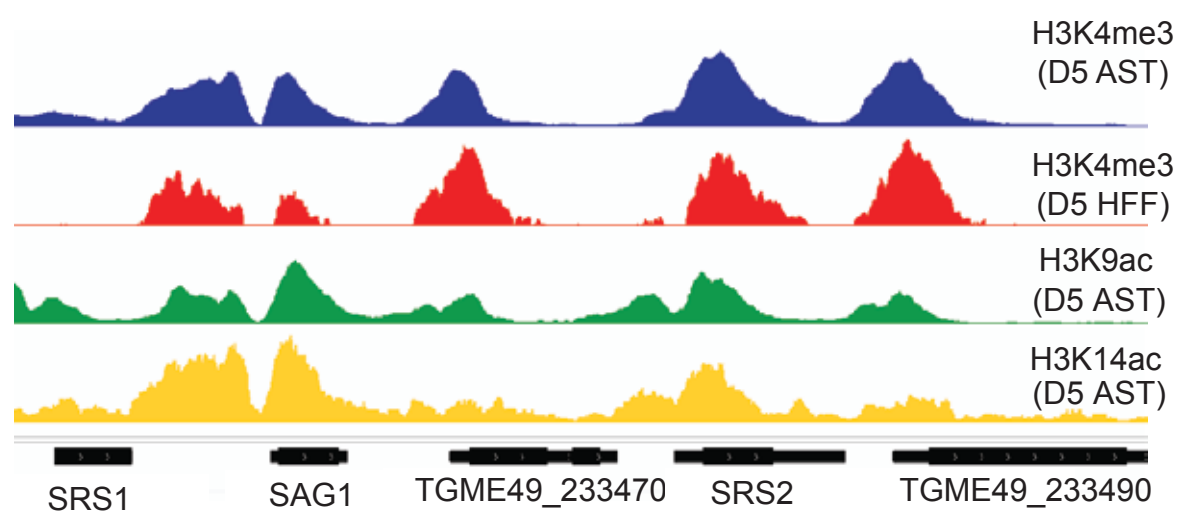

(B)

TSS ( $\pm$  1kb) - Consensus H3K4me3 (AST and HFF), H3K9ac, H3k14ac

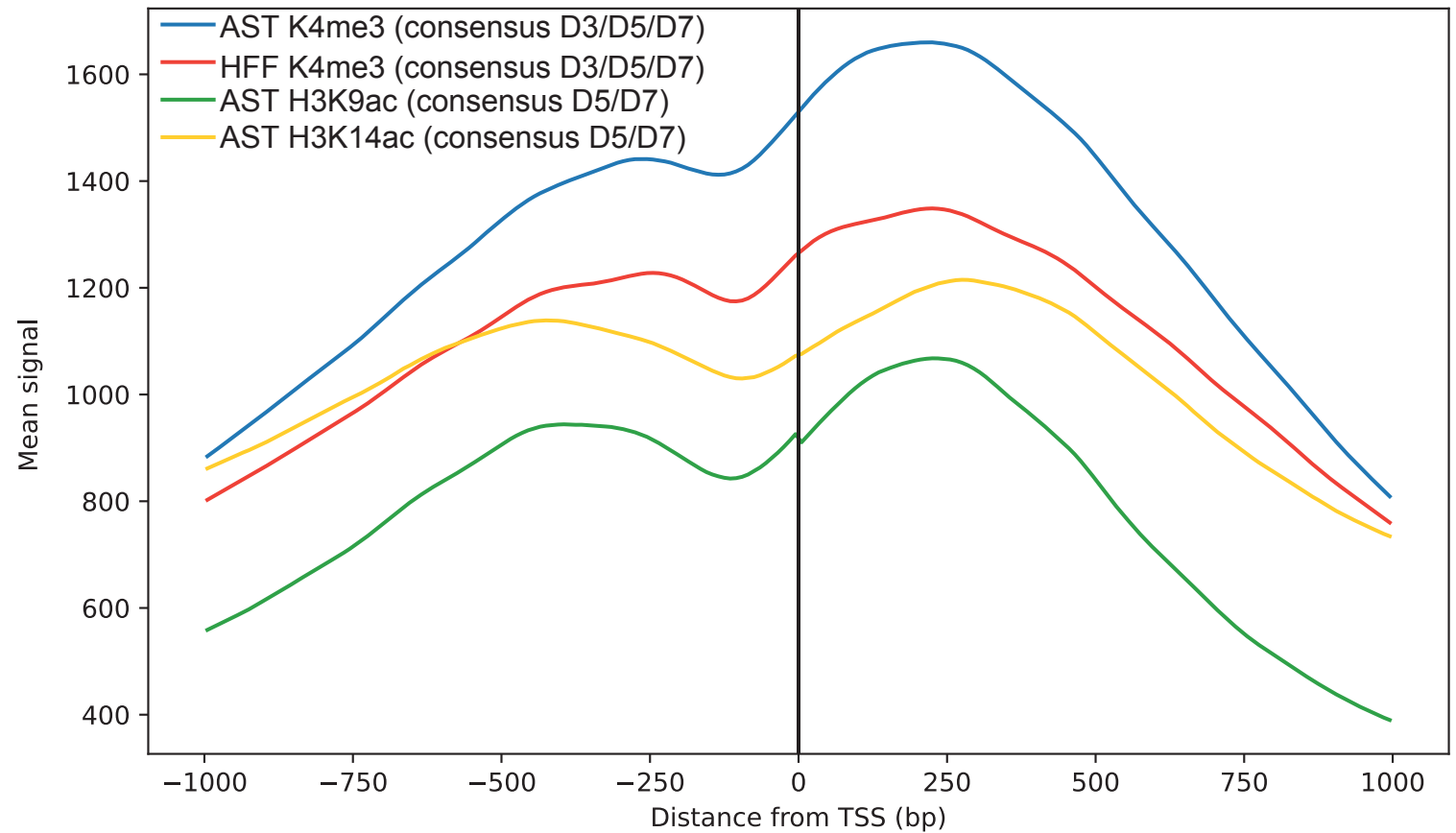

Supplement: Supplementary file 2 — Additional file 2: Figure S2. Genome-wide relationship between H3K4me3 and histone acetylation marks. (A) Spearman correlation analysis of promoter-proximal signal (±1 kb from annotated TSS) demonstrates strong concordance between H3K4me3, H3K9ac, and H3K14ac across genes. Correlation coefficients (ρ = 0.76–0.86) indicate substantial co-enrichment of active histone marks. (B) Aggregate TSS-centered profiles (±1 kb) show promoter-associated enrichment of H3K4me3 in both astrocytes (AST) and fibroblasts (HFF). H3K9ac and H3K14ac display similar promoter-centered distributions, consistent with active chromatin architecture. (C) Representative genome browser view illustrating co-localization of H3K4me3 and acetylation marks at actively transcribed loci, including SAG1. These results indicate that H3K9ac and H3K14ac broadly mirror H3K4me3 promoter enrichment patterns, supporting the use of H3K4me3 as the primary active chromatin mark for downstream analyses. [file 13072_2026_678_MOESM2_ESM.pdf]

H3K4me3 at SAG1 and SRS9 TSS ( $\pm 1$  kb) — Day 3 vs Day 5

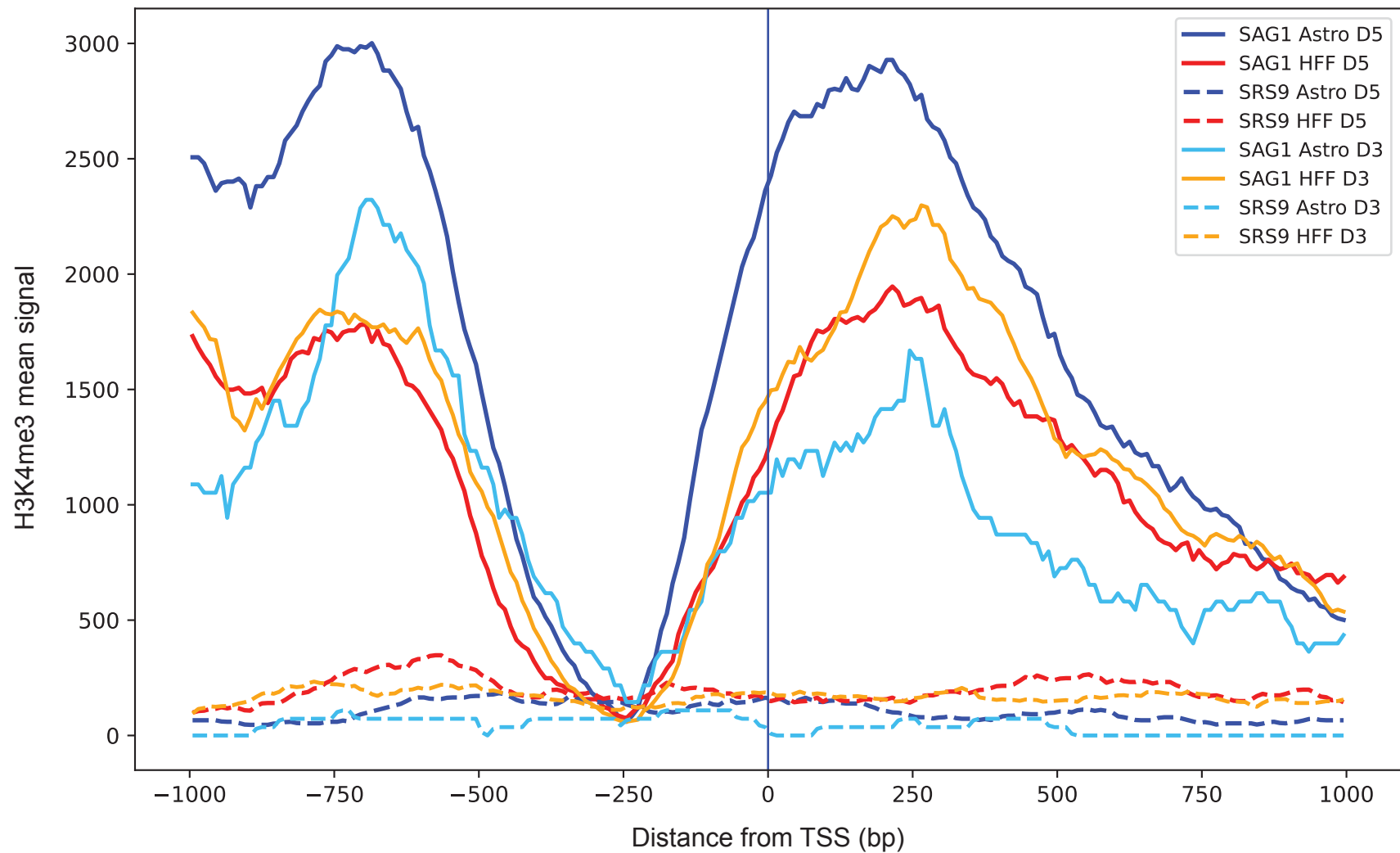

Supplement: Supplementary file 3 — Additional file 3: Figure S3. Promoter-associated H3K4me3 signal at SAG1 and SRS9 loci. TSS-centered H3K4me3 profiles (±1 kb) were extracted from deepTools matrices for SAG1 (TGME49_233460) and SRS9 (TGME49_320190) in astrocytes (AST) and fibroblasts (HFF) at Day 3 and Day 5. Solid lines represent SAG1 and dashed lines represent SRS9. SAG1 shows strong promoter-proximal H3K4me3 enrichment in both host environments, whereas SRS9 remains near baseline without host-dependent gain of promoter-associated H3K4me3. [file 13072_2026_678_MOESM3_ESM.pdf]

RNA Pearson Correlation Heatmaps

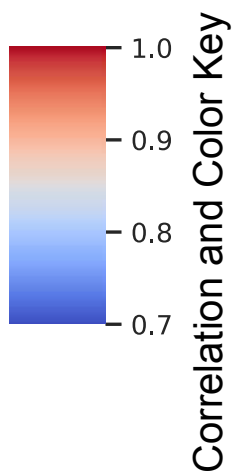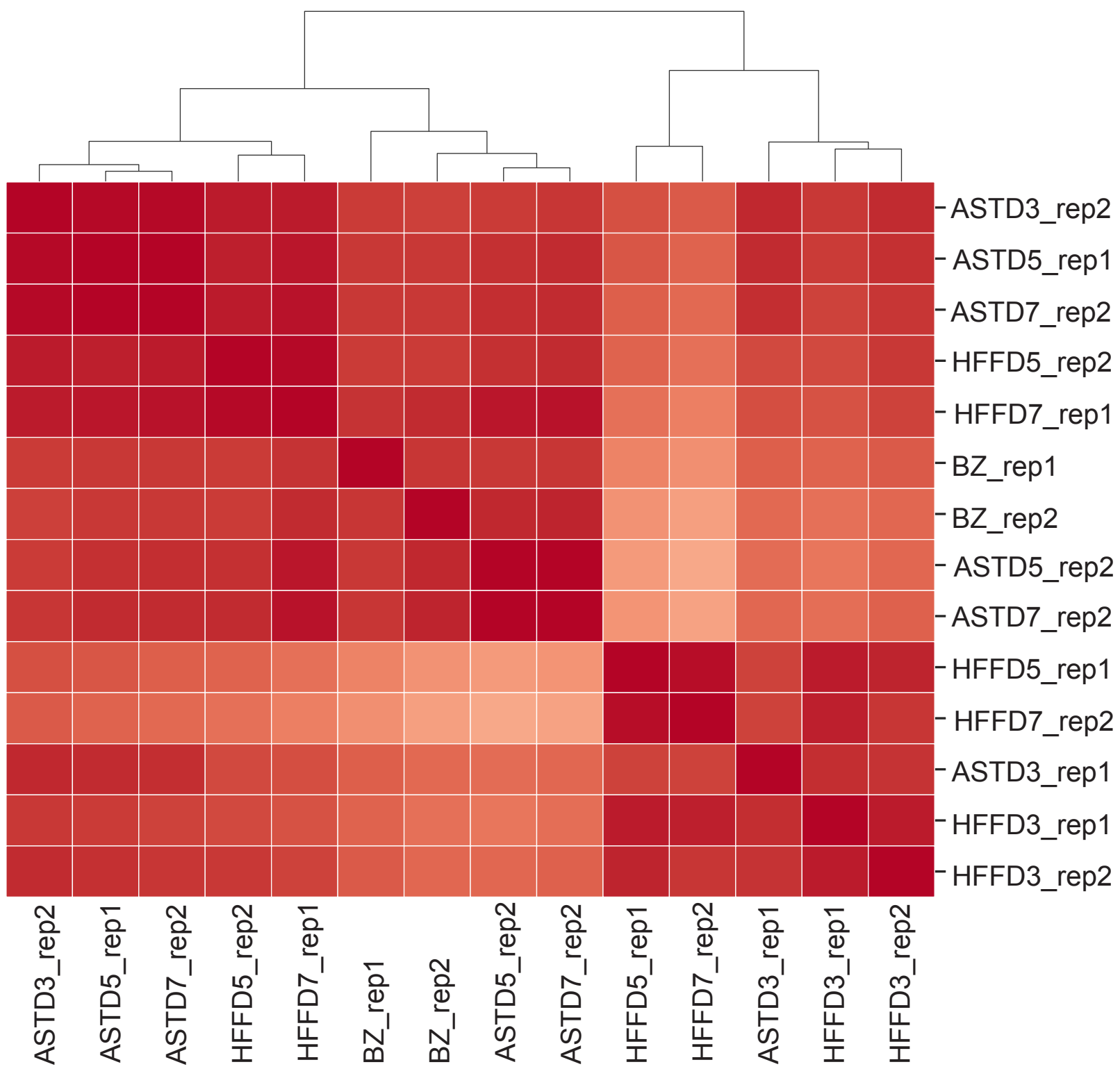

Supplement: Supplementary file 4 — Additional file 4: Figure S4. Pearson correlation heatmap of RNA-seq expression profiles across host conditions. Pairwise Pearson correlations for library size normalized read counts. All replicates exhibited strong agreement (r > 0.9), confirming high sequencing reproducibility. Samples clustered primarily by host cell type (AST vs HFF). [file 13072_2026_678_MOESM4_ESM.pdf]
